# The Treatment of Craniocervical Instability Using Regenerative Medicine Therapies: A Case Report

**DOI:** 10.7759/cureus.113501

**Published:** 2026-07-28

**Authors:** Hannah E Jones, Joseph A Ierulli, Christopher Williams, John Pitts

**Affiliations:** 1 Physical Medicine and Rehabilitation, Emory University, Atlanta, USA; 2 Physical Medicine and Rehabilitation, Emory University School of Medicine, Atlanta, USA; 3 Physical Medicine and Rehabilitation, Regenexx Cayman, George Town, CYM; 4 Physical Medicine and Rehabilitation, Interventional Orthopedics and Regenerative Medicine, Interventional Orthopedics of Atlanta, Atlanta, USA; 5 Interventional Pain Management, Centeno-Schultz Clinic, Denver, USA

**Keywords:** craniocervical instability, craniocervical junction, dextrose prolotherapy, orthobiologic medicine, platelet-rich plasma/prp

## Abstract

Craniocervical instability (CCI) is a disorder caused by weakness of the stabilizing ligaments of the craniocervical junction, leading to abnormal motion between the skull and upper cervical spine. CCI can present with any combination of the following symptoms: cervical neck pain, dizziness, autonomic dysfunction, migraines, and radiculopathy/myelopathy. CCI is often associated with connective tissue disorders. We present the case of a 51-year-old woman with a history of rheumatoid arthritis and chronic neck pain, migraines, vertigo, and autonomic dysfunction who underwent a series of regenerative injection treatments alongside postural and chiropractic therapy. Symptomatic and structural improvement was achieved, suggesting a possible role for regenerative therapies in the treatment of CCI. However, further studies are needed to investigate the effects of regenerative therapies on CCI severity. Our primary objective in presenting this case is to continue the conversation regarding the treatment of CCI with regenerative therapies and to spur further research on the topic.

## Introduction

Craniocervical instability (CCI) is a disorder characterized by excessive motion at the craniocervical junction (the point where the skull and cervical spine meet), resulting from insufficiency of the ligamentous stabilizers connecting the occiput, atlas (C1), and axis (C2). The alar, transverse, accessory, tectorial membrane, capsular, and other supporting ligaments function together to maintain cervical spine stability while permitting physiologic motion. Injury, inflammatory arthropathy, inherited connective tissue disorders, or trauma may compromise these stabilizing structures, leading to abnormal biomechanics and, in severe cases, to deformation or compression of the cervicomedullary junction, upper spinal cord, vertebral arteries, or lower cranial nerves [1-2].

The true prevalence of CCI in the general population remains unknown because standardized diagnostic criteria have not been universally adopted. However, CCI is increasingly recognized in patients with hereditary connective tissue disorders, particularly hypermobile Ehlers-Danlos syndrome, as well as in patients with rheumatoid arthritis, cervical trauma, and whiplash-associated disorders. Atlantoaxial instability has been reported in approximately 25-65% of patients with longstanding rheumatoid arthritis, although only a subset of these patients develop clinically significant neurologic compromise [3-4].

Because the craniocervical junction contains numerous neurologic and vascular structures within a confined anatomic space, ligamentous instability may produce a broad spectrum of symptoms, including occipital headaches, neck pain, dizziness, disequilibrium, visual disturbance, bulbar dysfunction (difficulty speaking, chewing, or swallowing), dysautonomia (instability in blood pressure and heart rate regulation), cervical radiculopathy (radiating “shock-like” neck pain), and muscular weakness. In advanced cases, deformation or compression of the cervicomedullary junction may produce the constellation of the above-mentioned neurologic findings known as cervicomedullary syndrome [2].

## Case presentation

We present the case of a 51-year-old woman with a history of rheumatoid arthritis, chronic neck pain, anterior/posterior cervical muscular tightness, and migraines. Symptoms began in 2004 with migraines and pain at the base of the neck, extending into the shoulders. In 2015, she sought treatment with unspecified chiropractic adjustments (i.e., manual manipulation), which relieved many of her symptoms. In 2019, a traditional chiropractic adjustment resulted in dizziness and bilateral arm numbness. Two months later, in November 2020, she was at a physical therapy visit when she felt that her upper cervical discs had shifted, leading to another ER visit for vertigo and head pressure.

**Figure 1:**
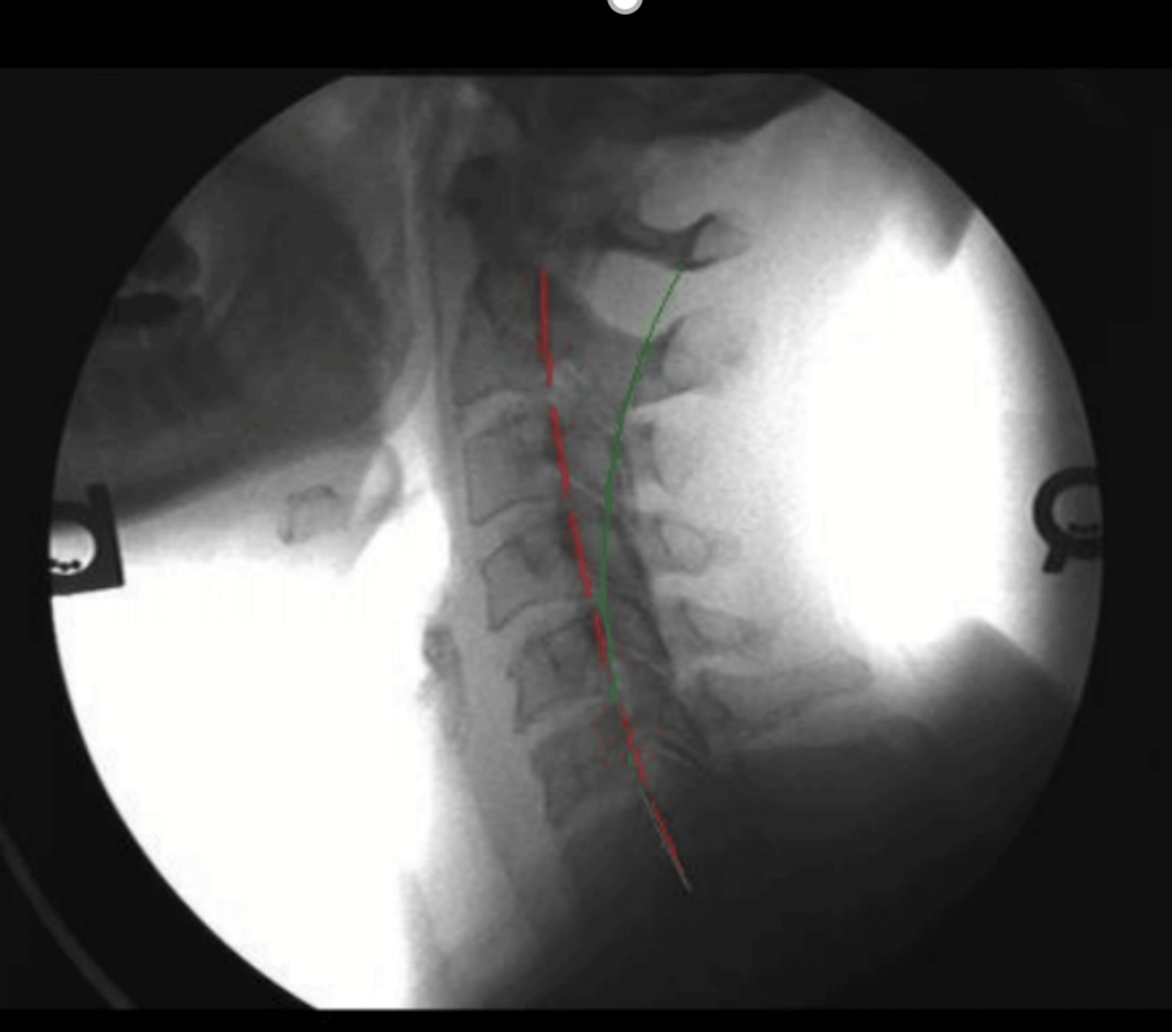
DMX image - 1 Lateral view with red dotted line showing the patient's curvature in a neutral position along the posterior longitudinal ligaments. The green line represents the normal spine position and normal curvature in a neutral position DMX: digital motion X-ray

**Figure 2:**
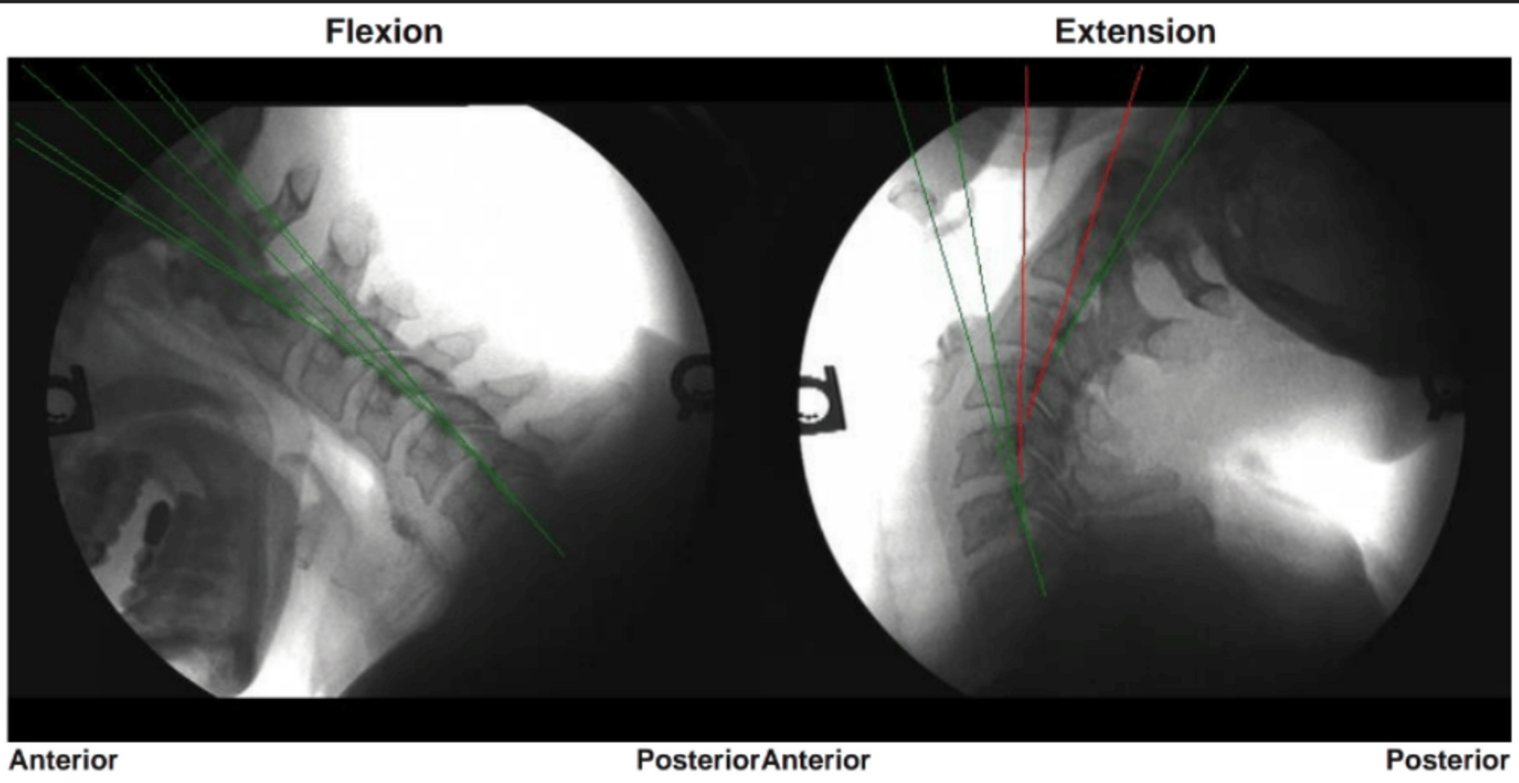
DMX image(s) - 2 Flexion and extension positioning of the cervical spine, with red lines showing abnormal alignment and green lines demonstrating normal alignment DMX: digital motion X-ray

**Figure 3:**
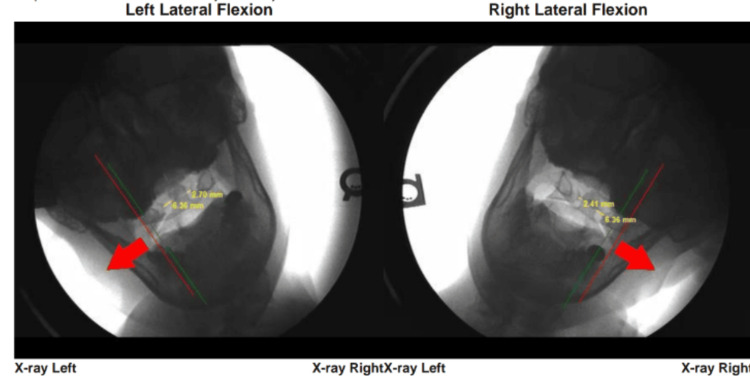
DMX image(s) - 3 Lateral bending of the cervical spine in the AP view demonstrating abnormal overhang of the C1 lateral mass on C2 dynamically when bending the neck laterally from left to right from the neutral position. The red line represents the position of the atlas lateral mass in the side-bending position. The green line represents the position of the axis superior articular process. Shifting of the red line greater than 3.0 mm from the green indicates probable damage to the alar and accessory ligaments DMX: digital motion X-ray

Over the next couple of weeks, she began to have autonomic dysregulation with short exercise and went to the emergency department, where she was diagnosed with orthostatic tachycardia. Vertigo and headaches continued, and an MRI in 2021 showed degenerative disc disease at C3-4, C4-5 with left C4-5 facet arthropathy causing mild neuroforaminal canal stenosis. She presented to an outpatient interventional orthopedic practice in 2021. Digital motion X-ray (DMX) of her cervical spine revealed loss of lordosis by 52.6%, anterolisthesis suggestive of anterior longitudinal ligament (ALL) instability at C2 relative to C7 of 30.7mm, C1-2 lateral overhang with right lateral bending of 7.1 mm, which is highly abnormal (Figures 1-3, Tables 1-2). A CT of the cervical spine was largely unremarkable aside from C4-5 facet joint arthropathy with normal cervical alignment.

**Table 1:** Left cervical bending WNL: within normal limits

Global analysis	Normal levels	Patient values	Clinical significance	
C0-C1 Lat. Flex. angle	< 5.0°	5.1°	Possible instability	
C1-C2 Lat. Flex. angle	< 5.0°	4.9°	WNL	
C2-C3 Lat. Flex. angle	< 20.0°	5.1°	WNL	
C1-C2 overhang	< 3.0 mm	Left 3.6 mm	Possible instability	
C2 Axial apinous rotation	< 10.0°	Right 29.6°	Possible instability	

**Table 2:** Right cervical bending WNL: within normal limits

Global analysis	Normal levels	Patient values	Clinical significance	
C0-C1 Lat. Flex. angle	< 5.0°	1.9°	WNL	
C1-C2 Lat. Flex. angle	< 5.0°	2.6°	WNL	
C2-C3 Lat. Flex. angle	< 20.0°	0.2°	WNL	
C1-C2 overhang	< 3.0 mm	right -7.1 mm	Possible instability	
C2 Axial spinous rotation	< 10.0°	left 25.9°	Possible instability	

In December 2021, the patient underwent injections to the bilateral intra-articular facet joints (C0-1, C1-2, C2-3, and C-4), posterior cervical ligaments (nuchal ligament, posterior atlanto-occipital membrane, supraspinous, and interspinous ligaments), and anterior longitudinal ligament with platelet lysate (PL) and platelet-rich plasma (PRP). The procedures were performed by the treating physician (who is also an author of this report). Due to lack of insurance coverage for these specific procedures/injections, the interventions were completed on an out-of-pocket, self-pay basis.

**Figure 4:**
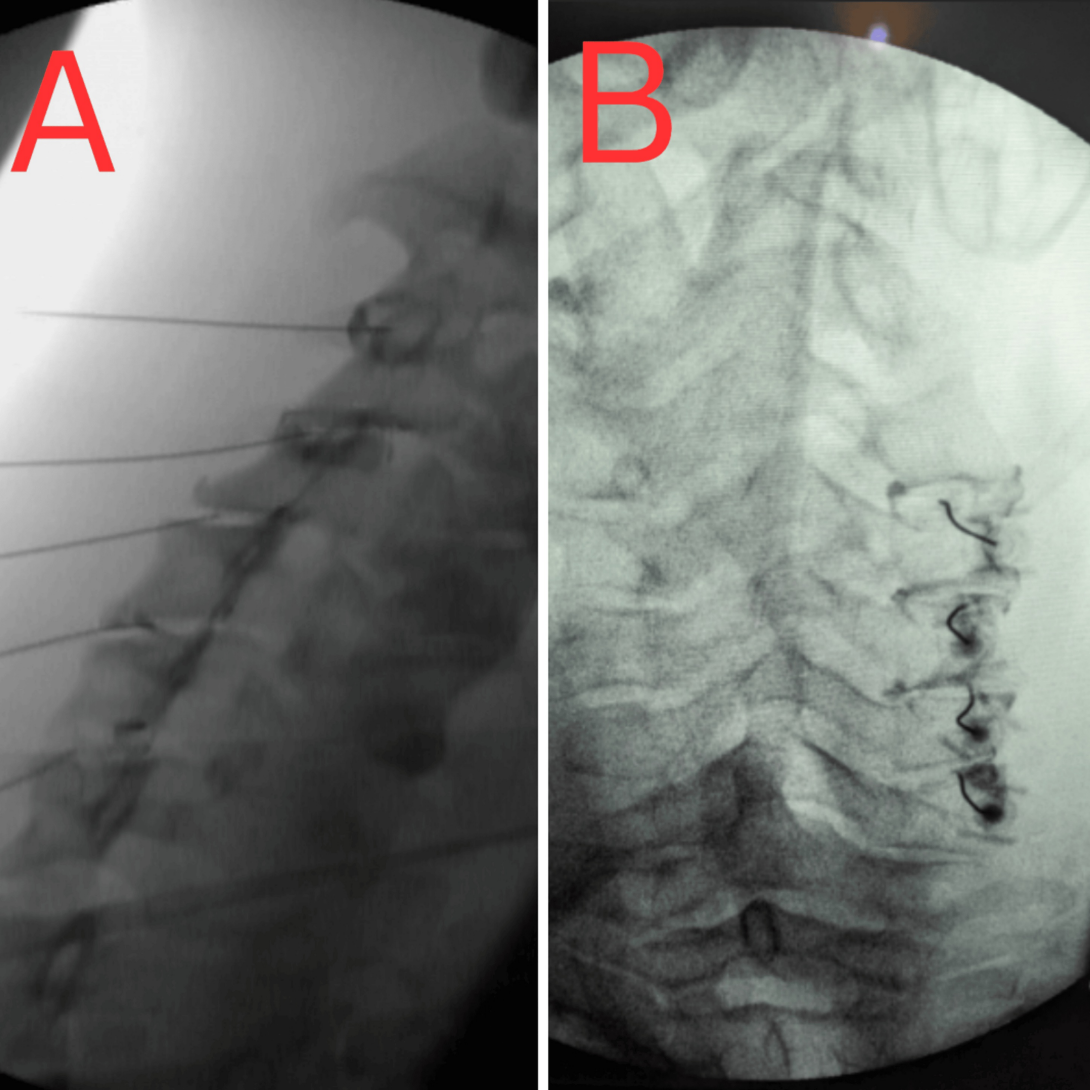
Fluoroscopic-guided intra-articular facet injections in the cervical spine utilizing a contralateral oblique (A) and anteroposterior views (B)

**Figure 5:**
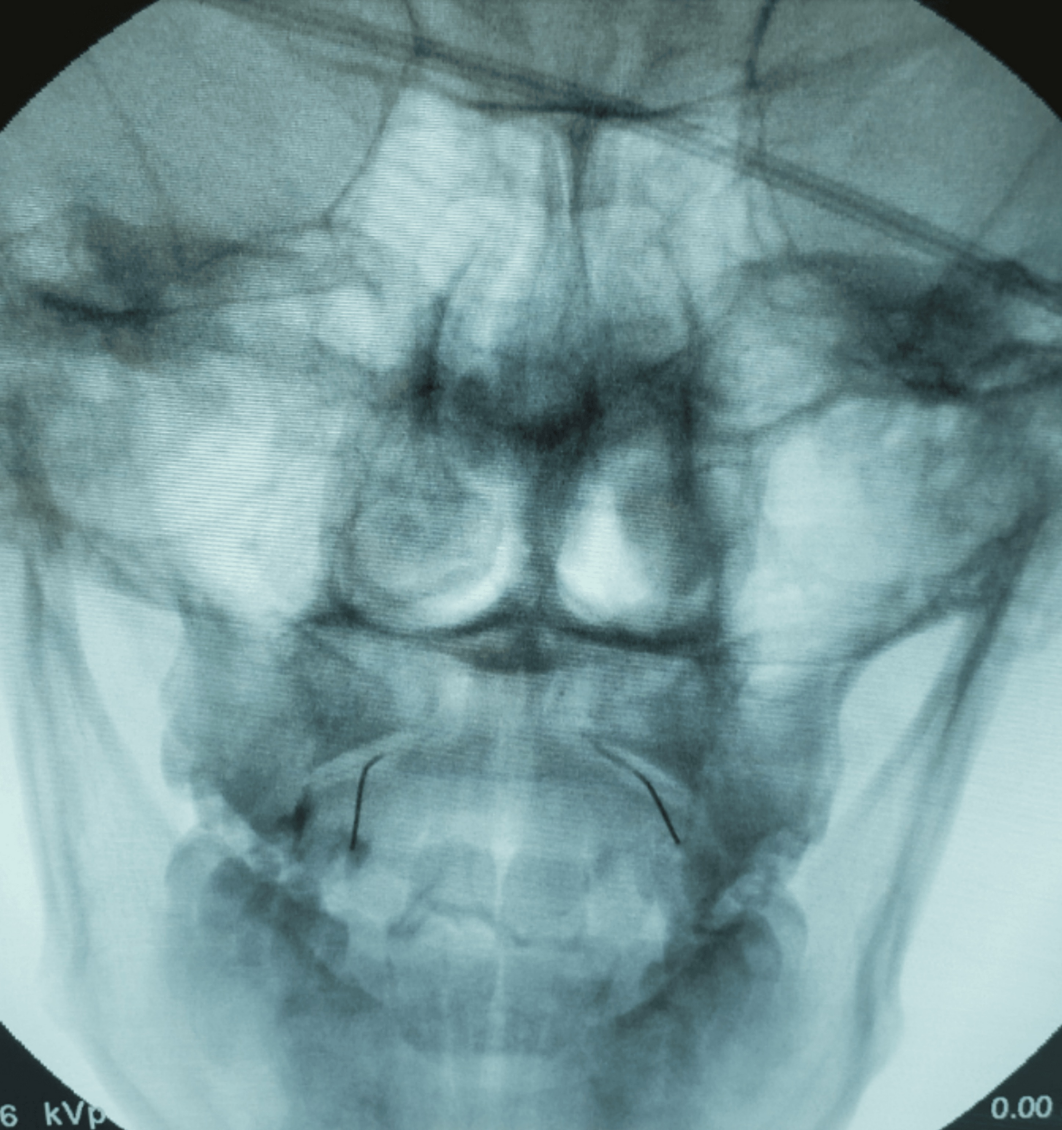
Anteroposterior view of fluoroscopic-guided intra-articular C1-C2 facet joint injections

**Figure 6:**
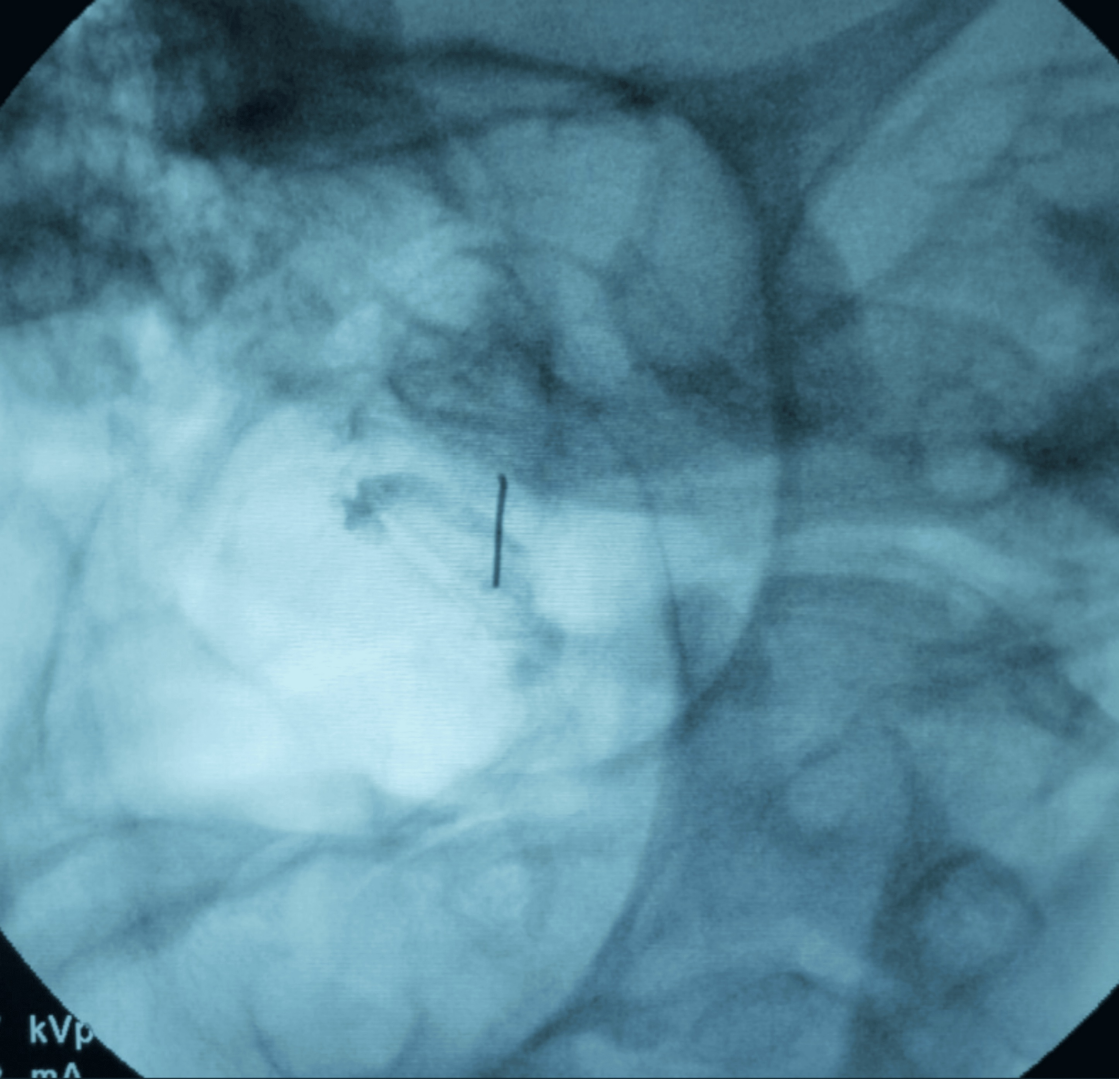
Anteroposterior view of C0-C1 intra-capsular injection on the left side

**Figure 7:**
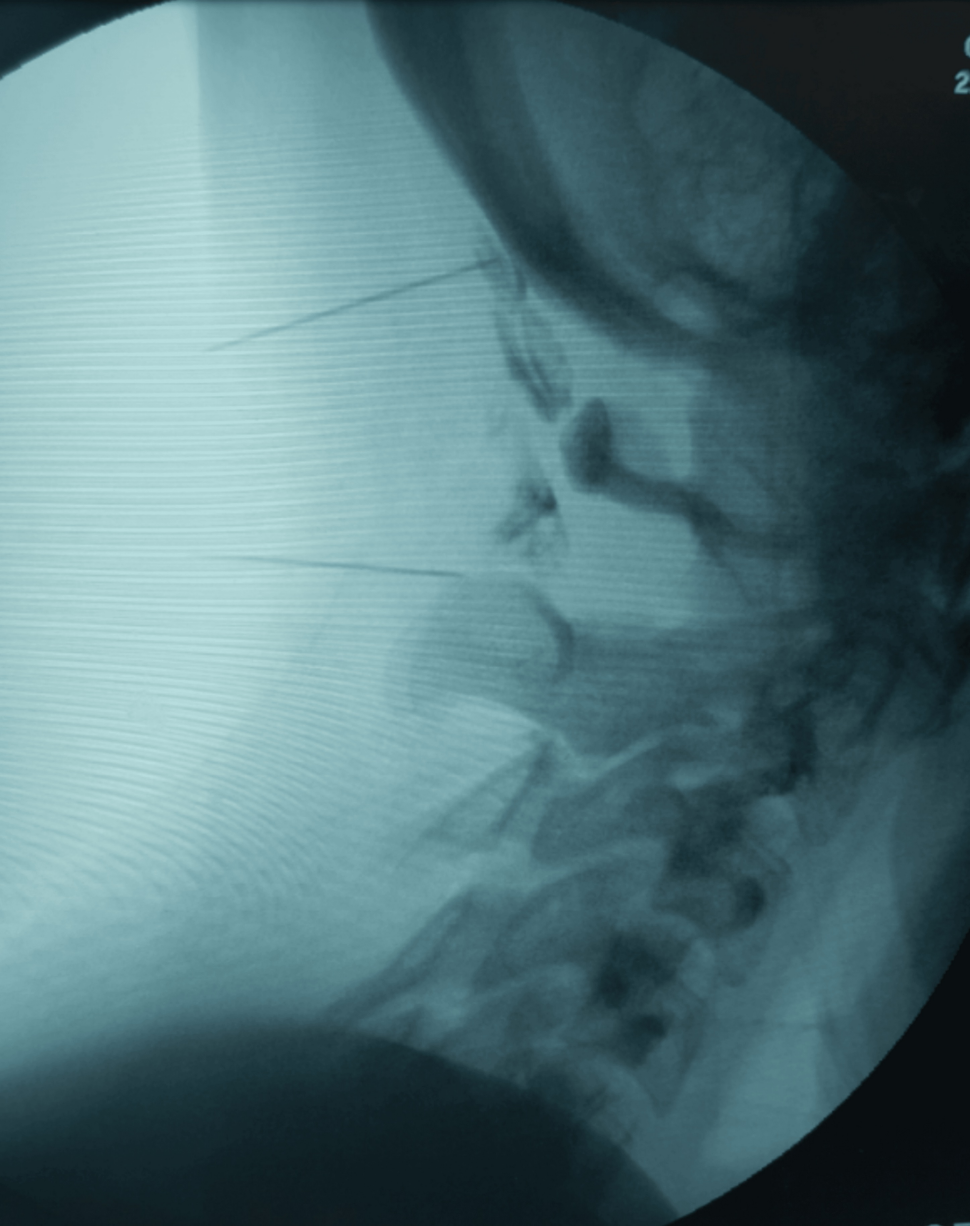
Lateral view of fluoroscopic-guided posterior spine ligamentous injections with contrast confirmation

**Figure 8:**
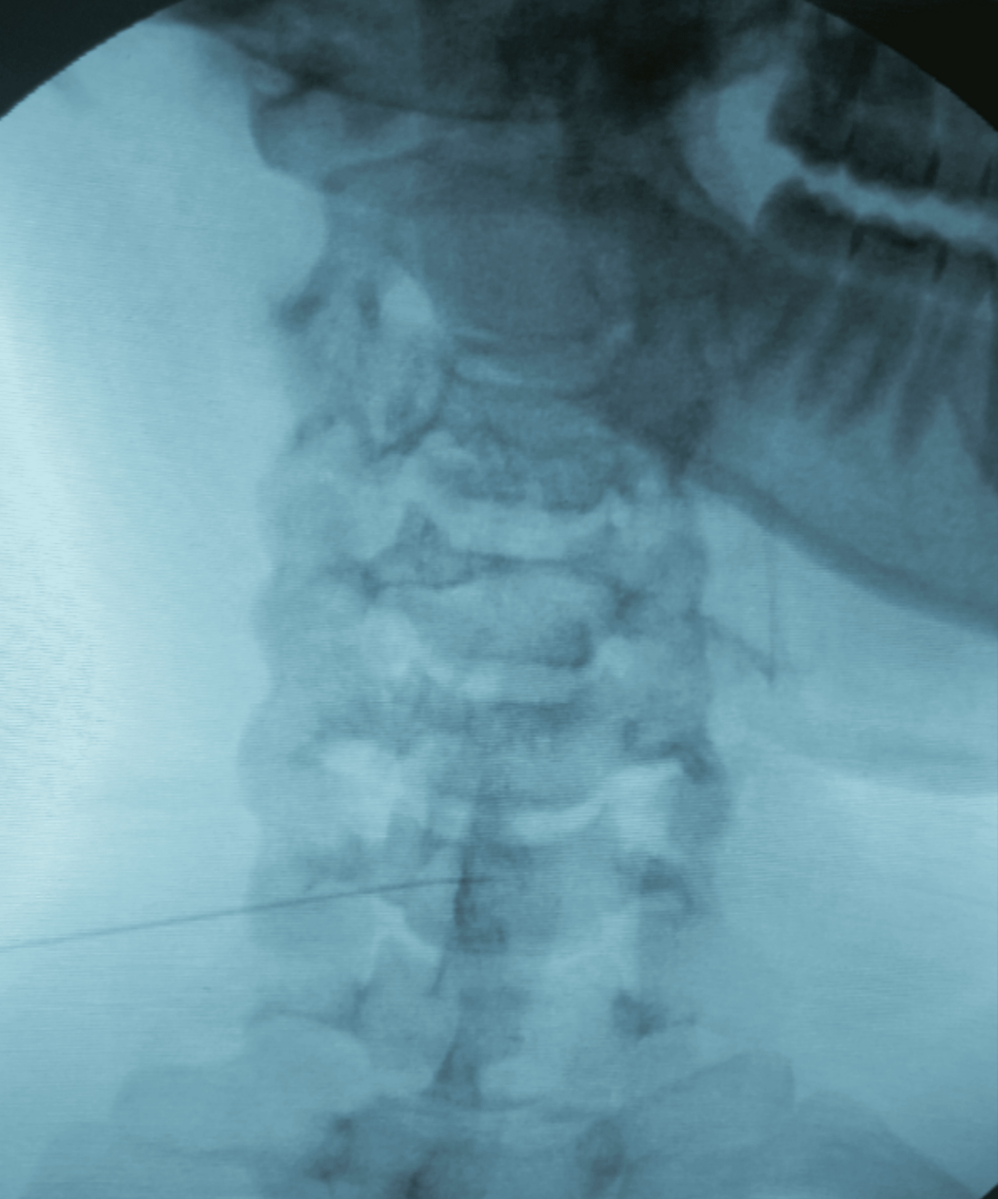
Anteroposterior view of fluoroscopic-guided anterior longitudinal ligament

**Figure 9:**
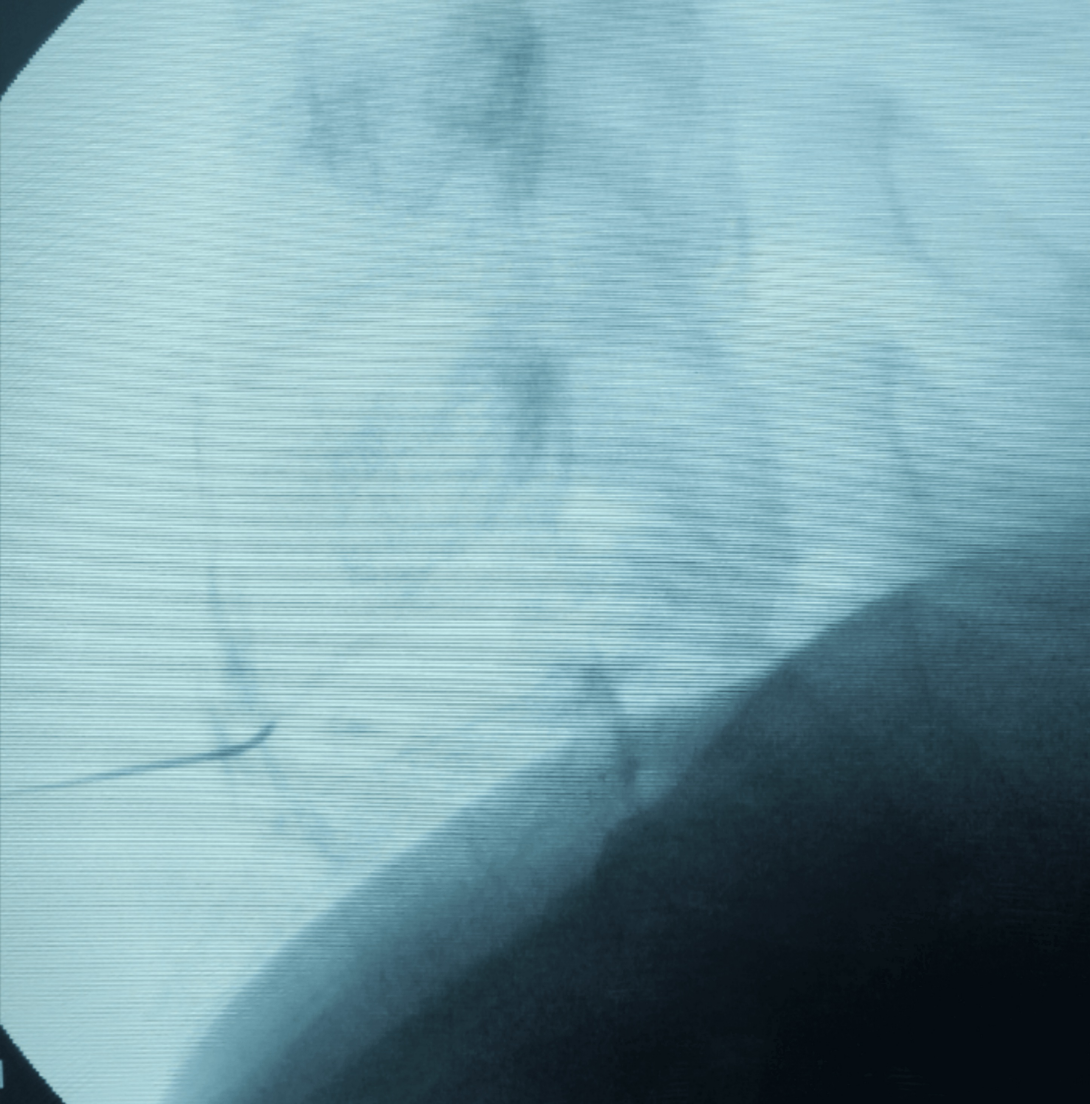
Lateral view of fluoroscopic-guided anterior longitudinal ligament

**Figure 10:**
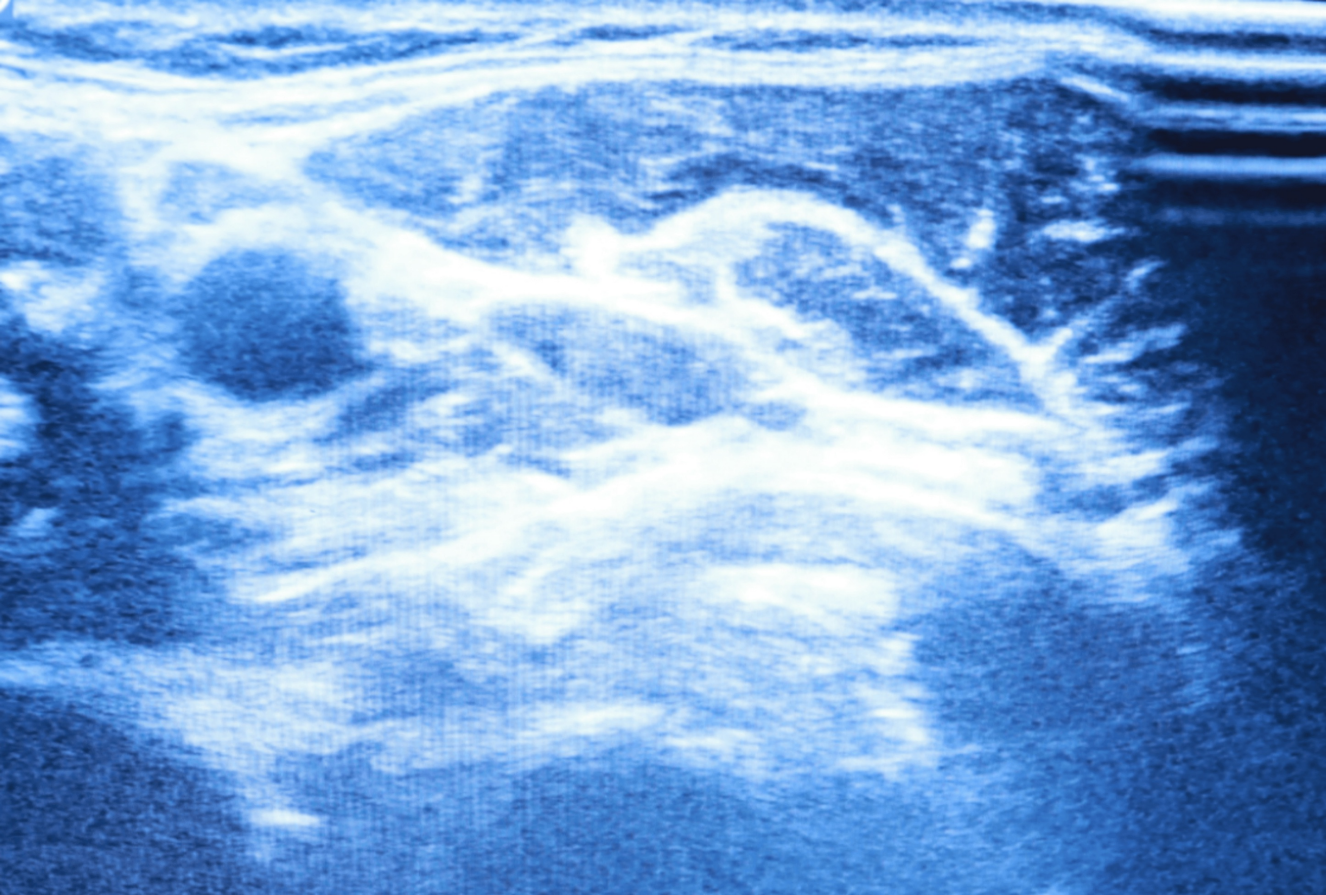
Ultrasound-guided needle trajectory for the anterior longitudinal ligament

Fluoroscopy images of the injection procedure(s) are shown in Figures 4-10. The PL and PRP were prepared as previously described in the literature [8]. In brief, 180-200 mL of whole blood was collected, and a two-spin centrifugation technique was utilized for the preparation of concentrated leukocyte- and red blood cell-poor PRP. A freeze-thaw method with additional centrifugation of the platelet pellet was used to prepare the PL. PL was injected into the upper cervical facet joints (i.e., C0-1 and C1-2 facet joints), whereas PRP was injected into the lower joints (i.e., C2-C4 facet joints) and the anterior longitudinal ligament. Dextrose (15%) prolotherapy was added to the supraspinous and interspinous ligaments. The patient also underwent a baseline CBC, which revealed a platelet count of 254,000/µL. Based on an expected platelet recovery rate of 80%, a table was constructed showing the estimated platelet dose injected into the treated structures (Table 3).

**Table 3:** Dosing sheet for calculating the estimated platelet dose Estimated platelet dose calculation: a platelet concentration of 254,000 platelets/µL corresponds to 254 × 10⁶ platelets/mL. Assuming an 80% platelet recovery, the estimated platelet concentration is 254 × 10⁶ × 0.80 = 200 × 10⁶ platelets/mL. The estimated platelet dose delivered to each injected structure was calculated by multiplying this value by the volume (mL) of biologic injected into that structure PRP: platelet-rich plasma; PL: platelet lysate; IAF: intra-articular facet; ALL: anterior longitudinal ligament; SS/IS = supraspinous/interspinous

Baseline platelet dose	Whole blood volume collected (mL)	Biologic utilized	Structure(s) injected	Volume of biologic injected (mL)	Estimated platelet dose	Total estimated platelets injected
254,000/µL	156	PL	C0-1, C1-2 IAF Joints	4	7.9 x 10^9^/mL	32 x 10^9 ^
	59	PRP, PL	C2-3, C3-4 IAF joints	2, 2	3 x 10^9^/mL	12 x 10^9 ^
	11	PRP	ALL	1	2.2 x 10^9^/mL	2.2x 10^9 ^
	33	PL	SS/IS ligaments	4	1.65 x 10^9^/mL	6.6 x 10^9^
	Total: 259 mL					Total: 51.8 x 10^9^

**Figure 11:**
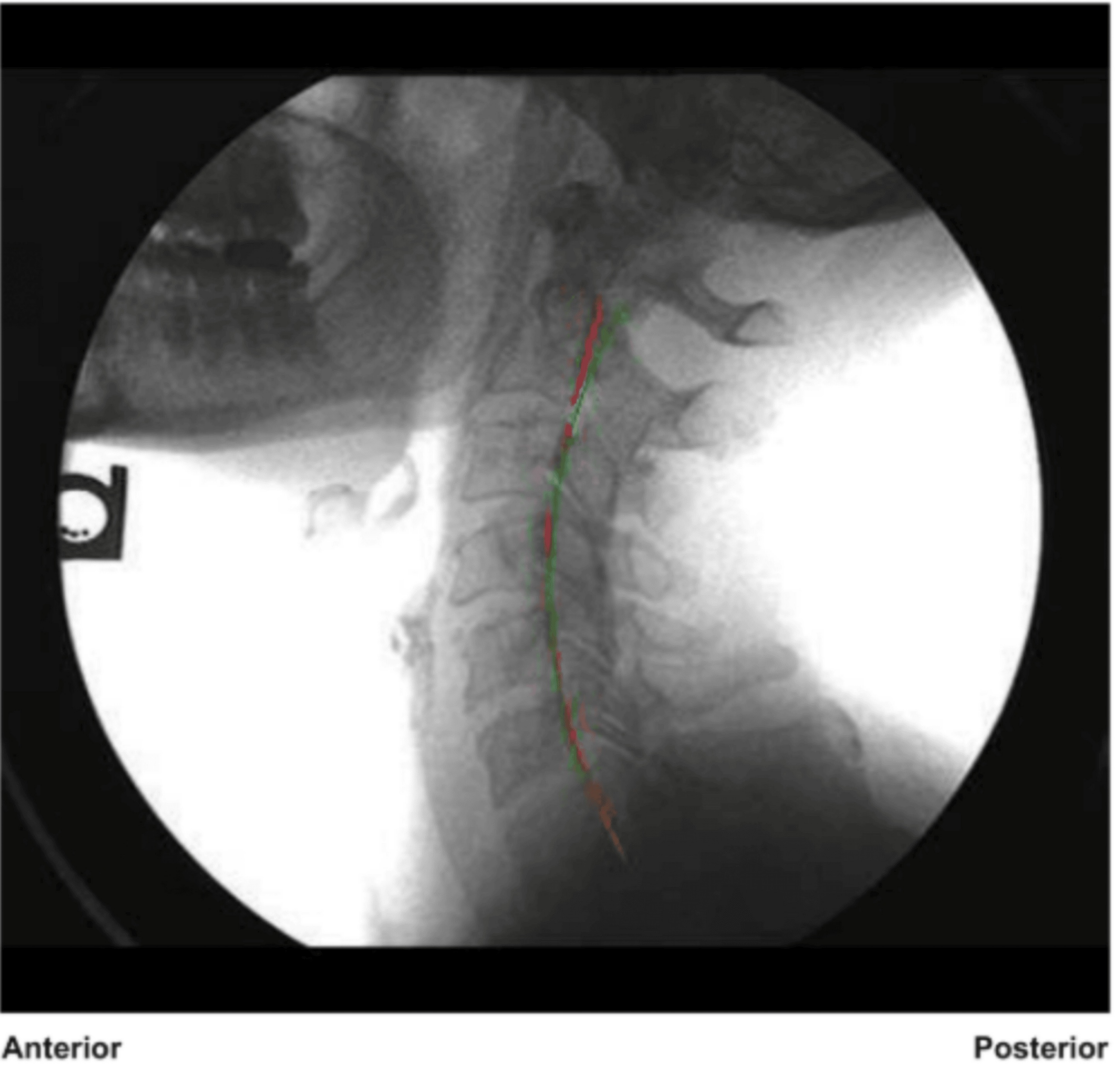
DMX image(s) - 4 Lateral view with a red dotted line showing the patient's curvature in a neutral position along the posterior longitudinal ligaments. The green line represents the normal spine position and normal curvature in a neutral position

**Figure 12:**
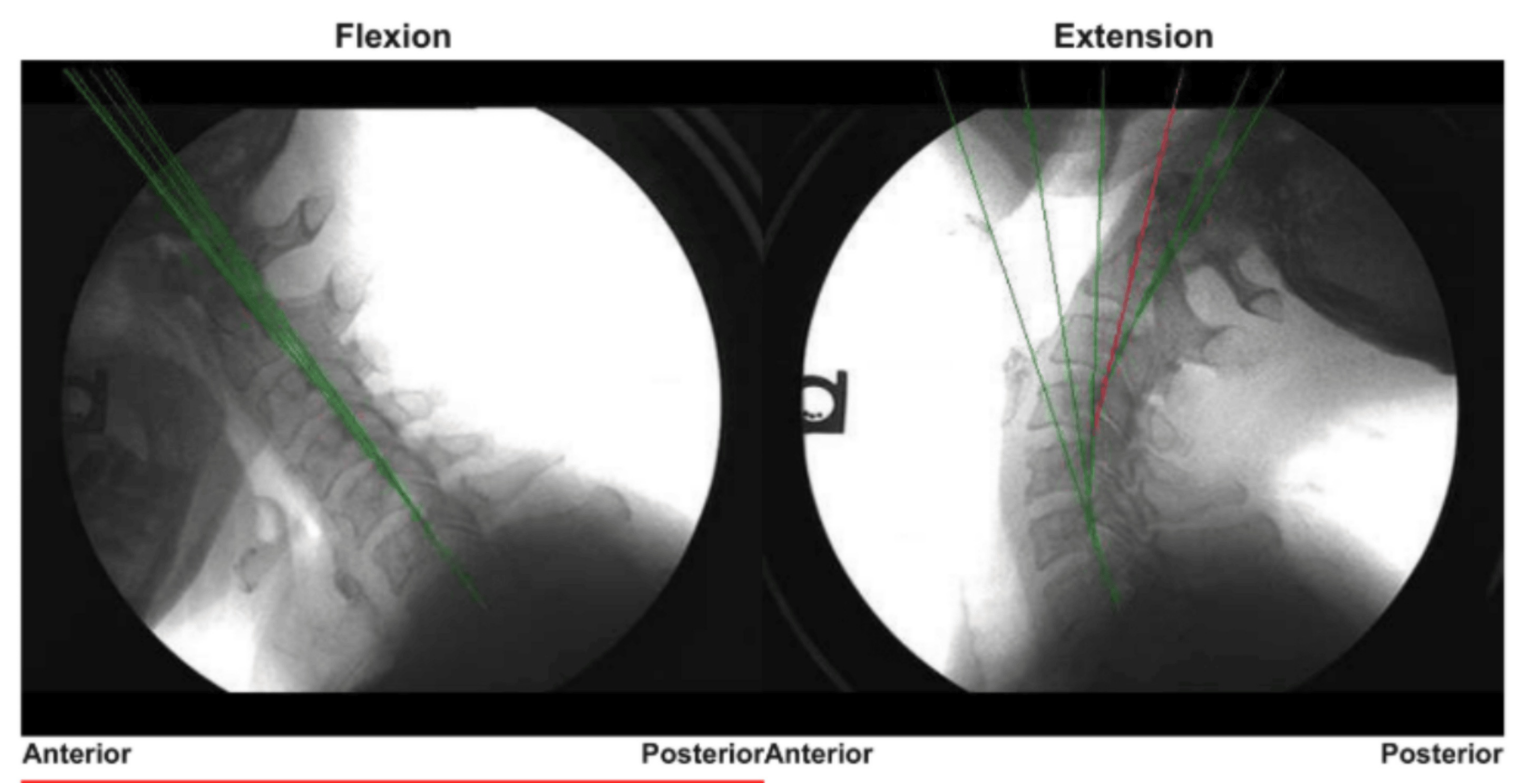
DMX image(s) - 5 Flexion and extension positioning of the cervical spine with red lines showing abnormal alignment and green lines demonstrating normal alignment

**Figure 13:**
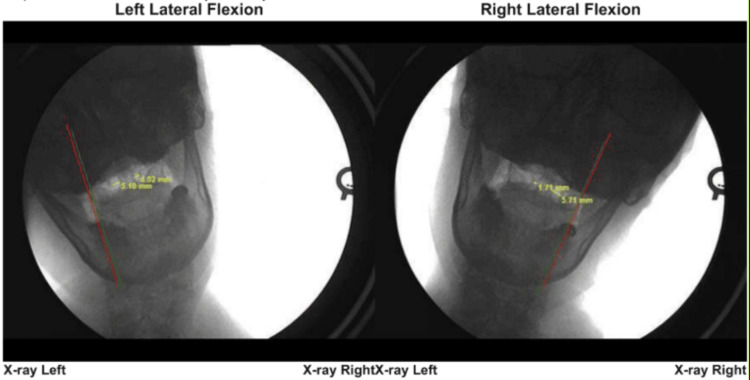
DMX image(s) - 6 Lateral bending of the cervical spine in the AP view demonstrating no abnormal overhang of the C1 lateral mass on C2 dynamically when bending the neck laterally from left to right from the neutral position. The red line represents the position of the atlas lateral mass in the side-bending position. The green line represents the position of the axis superior articular process. Shifting of the red line greater than 3.0 mm from the green indicates probable damage to the alar and accessory ligaments

Due to continued symptoms and instability noted on DMX imaging, similar injection procedures were completed 10 more times over the course of four years at various timing intervals. No procedural complications occurred. Also during this time, the patient continued physical therapy working on cervical musculature strengthening and stretching exercises. She also started Egoscue therapy to help with postural corrections and stability of her spine. DMX imaging continued to improve after each injection, ultimately seeing normalization of C1-C2 overhang with remaining mild anterolisthesis at C2 relative to C7 (15.8mm), and near complete re-establishment of lordosis with a loss of lordosis only 25.2% from the expected normal (Figures 11-13, Tables 4-5). Despite the possibility of variability in measurements and patient positioning across individual DMX reports, there was a general trend toward structural improvement over the course of treatment. Table 6 provides a detailed timeline of interventions, symptoms, and imaging findings.

**Table 4:** Left cervical bending WNL: within normal limits

Global analysis	Normal levels	Patient values	Clinical significance	
C0-C1 Lat. Flex. angle	< 5.0°	1.8°	WNL	
C1-C2 Lat. Flex. angle	< 5.0°	1.9°	WNL	
C2-C3 Lat. Flex. angle	< 20.0°	2.9°	WNL	
C1-C2 overhang	< 3.0 mm	Left 1.9 mm	WNL	
C2 Axial spinous rotation	< 10.0°	Right 23.8°	Possible instability	

**Table 5:** Right cervical bending WNL: within normal limits

Global analysis	Normal levels	Patient values	Clinical significance	
C0-C1 Lat. Flex. angle	< 5.0^°^	1.6^°^	WNL	
C1-C2 Lat. Flex. angle	< 5.0^°^	0.3^°^	WNL	
C2-C3 Lat. Flex. angle	< 20.0^°^	1.9^°^	WNL	
C1-C2 overhang	< 3.0 mm	Right -1.1 mm	WNL	
C2 Axial spinous rotation	< 10.0^°^	Left 17.0°	Possible instability	

**Table 6:** Treatment timeline DDD: degenerative disc disease; PT: physical therapy; DMX: digital motion X-ray; ARA: absolute rotational angle; ALL: anterior longitudinal ligament; PLM: platelet lysate matrix; SCP: super-concentrated platelets

Timeline	Symptoms	Imaging	Intervention	Response
2004	Symptoms began with migraines, pain at base of neck, tightening of upper cervical musculature	----	----	----
July 2021- September 2021	Severe occipital pain, neck and shoulder tightness, dependence on cervical collar	Cervical MRI with DDD, C3-C4 degeneration, C4-C5 degeneration, left C4-C5 facet arthropathy, mild neuroforaminal stenosis	Cervical chiropractic adjustment	Acute onset of upper extremity numbness, tingling, and dizziness
November 2, 2021	Head pressure, vertigo, orthostatic tachycardia, persistent cervical instability sensation, severe headaches	Cervical XR with 53% reduction in overall cervical lordosis compared with the ideal measurement (ARA C2-C7 −19.9° versus ideal −42°) and substantial anterior translation of C2-C7 (30.7 mm)	Chin-tuck exercises with PT	Vertigo and severe neck pain
December 2021	Chronic neck pain, occipital pain, shoulder pain, neck tightness, exercise intolerance, vertigo, dizziness, recurrent headaches, perceived cervical instability	DMX imaging with cervical hypermobility, loss of cervical lordosis, hyperextension instability (C4-C6), bilateral C3-C4 facet joint gapping, C1-C2 lateral overhang, ligamentous laxity	1. Bilateral C0-C4 facet injections using PLM and dextrose prolotherapy -- 4cc 12x PLM into the C0-C2 facets. 2cc 10x SCP and 2cc 8x PLM into C3-4 facets 2. SS/IS ligaments -- 12.5% dextrose prolotherapy with 5x PLM (10cc). 3. C4 ALL -- 1cc 7x SCP. During this time, patient also continued going to PT	Patient was able to return to exercise and perform overhead presses (unable to do before the procedure) and also had reduction in cervical muscle tightness
July 2022	Chronic neck pain, occipital pain, shoulder pain, vertigo, dizziness, recurrent headaches, perceived cervical instability	DMX imaging with continued loss of cervical lordosis, upper cervical instability, facet joint laxity, anterior longitudinal ligament laxity, and C1-C2 lateral overhang	1. Bilateral C0-C1, C1-C2, C2-C3, C3-C4 IAF -- 4cc 12x PLM (into the C0-C2 facets), 2cc 10x SCP, 2cc 8x PLM 2. SS/IS ligaments -- 12.5% dextrose prolotherapy with 5x PLM (10cc) 3. C4 ALL -- 1cc 7x SCP. During this time, patient also continued going to PT	----
December 2022	Continued right-sided neck tightness, dizziness, and dysautonomia. Occasional flares with PT but less severe	DMX imaging with persistent cervical instability, facet joint laxity, ALL laxity, loss of normal cervical curvature	1. Bilateral C1-C2, C2-C3, C3-C4 IAF -- 2cc 12x PLM, 2cc 10x SCP, 2cc 8x PLM 2. SS/IS ligaments -- 12.5% dextrose prolotherapy with 6x PLM c. ALL at C5 -- 1cc 7x SCP	Reduced migraine frequency and vertigo resolved
May 2023	Mild pain and right-sided neck tightness, dizziness	C1-C2 lateral overhang improved to approximately 3.1 mm, down from greater than 7 mm with significant improvement in stability. Continued residual abnormalities including previously documented C3-C4 gapping	Posterior ligaments -- 12.5% dextrose prolotherapy, 7x PLM, ropivacaine	Reduced pain and muscular tightness, continued reduction in migraine severity/frequency, improved subjective sense of cervical stability. Based on patient and provider assessment, there was an 85% clinical improvement in symptoms
July 2023	----	----	----	Able to return to tennis, improved cervical posture noted by PT, ability to sleep without cervical collar
Agust 2023	----	DMX imaging with C2-C7 global cervical lordosis of approximately −32.8° to −32.9°, compared with an ideal −42°, C2-C7 anterior translation of approximately 21.1 mm, C7 posterior tangent angle of approximately 29.4°, improved curvature compared with the markedly reduced lordosis documented in 2021, although alignment not normalized completely	----	----
September 2023	Recurrence of migraines and continued dizziness	DMX imaging with right C1-C2 lateral overhang increased from approximately 3.1 mm to 5.4 mm and left-sided overhang measured approximately 1.7 mm	1. Bilateral C0-C1, C1-C2, C2-C3 IAF -- 12x PLM, 10x PLM 2. Posterior ligaments -- 8x SCP, 8x PLM	----
October 2023	Mild muscular tightness and dizziness	----	----	Returned to regular workouts and upper extremity exercises. Improved travel tolerance
January 2024	Flare in symptoms with reduced activity tolerance	DMX with mild interval worsening of right C1-C2 lateral overhang to approximately 5.7 mm	1. Bilateral C0-C1, C1-C2, C2-C3 IAF -- 12x PLM, 10x PLM 2. Posterior ligaments -- 8x SCP, 8x PLM	----
March 2024	----	----	----	Resolution of dizziness
May 2024	Continued absence of dizziness, tolerating PT well	DMX with improved cervical lordosis. Stable to slightly improved C1-C2 overhang. Improved anterior longitudinal ligament laxity	----	----
July 2024	----	----	1. Bilateral C0-C1, C1-C2, C2-C3 IAF -- filtered 12x PLM, 10x PLM 2. Posterior ligaments -- 8x PLM	----
August 2024	Continued improvement with less muscular tension	----	Continuing PT	----
November 2024	Tight anterior cervical musculature and inability to lie on right side	DMX imaging with continued loss of lordosis, C3-C4 and C4-C5 facet/capsular gapping, C1-C2 lateral-bending measurements of approximately 3.3 mm on the left and 4.3 mm on the right, persistent C2 rotational instability	----	Return to hiking and frequent travel with no issues
December 2024	----	----	1. Right C3-C6, Left C2-C7 IAF -- 2x PLM, 8x PLM 2. Posterior ligaments -- 8x PLM, dextrose	----
March 2025	----	----	1.Bilateral C0-C1, C1-C2, C2-C3, C3-C4, C4-C5, C5-C6, C6-C7 IAF -- 12x PLM, 8x PLM, 15% dextrose prolotherapy ropivacaine 2. Posterior ligaments -- 10x PLM, 20% dextrose prolotherapy, ropivacaine, 8x PLM	----
June 2025	----	DMX imaging improvements: normalization of C1-C2 lateral overhang. Continued improvement of upper cervical alignment. Residual abnormalities: mild C3-C4 facet gapping. Persistent C2 rotational instability	Began Egoscue therapy	
December 2025	Migraines with lateral head movement	DMX imaging with near-complete restoration of normal cervical lordosis, complete resolution of C1-C2 overhang. Remaining abnormalities: mild ALL laxity at C4-C5, mild bilateral C3-C4 capsular gapping, residual C2 rotational instability	----	Improved headaches, dizziness, posture, and ability to sleep on right side

## Discussion

This case demonstrates the potential role of regenerative injection therapies, including PRP, PL, and prolotherapy, in the treatment of cervical spine dysfunction associated with CCI. While the current literature on orthobiologic treatments for CCI remains limited, emerging evidence supports the broader application of these therapies in spinal instability and degenerative cervical conditions. Diagnosis of CCI is challenging due to the presenting symptoms being common in the general population and often resulting from causes other than CCI, as well as the broad overlap with other etiologies and the lack of clinical guidelines for a standardized assessment and diagnostic approach. The symptoms used for diagnosis can also be seen as part of post-concussive syndrome after a traumatic head injury. Additionally, autonomic and proprioceptive changes can be seen in patients with EDS without CCI.

Therefore, even in a patient population where CCI is more common, the above-mentioned symptoms may not be explained by CCI alone [5]. The clinical exam for evaluating CCI consists of a complete neurological exam, range of motion assessment, Spurling’s test, and the extension-rotation test. CCI cannot typically be diagnosed on static imaging such as a routine supine cervical MRI or routine cervical X-rays. Since instability is a dynamic problem, movement-based imaging is required to provide objective evidence of instability, which is not routinely ordered by most physicians.

Based on the current literature, DMX/video fluoroscopy, upright flexion-extension MRI, and rotational CT scans are the imaging modalities most likely to diagnose CCI in selected patients when certain measurements exceed established norms. DMX imaging measurements are performed by capturing a live fluoroscopic video at 30 frames per second while a patient actively moves the joint(s) being evaluated. This is followed by automated software-based mensuration analysis to obtain values such as C1-C2 lateral overhang. This differs from conventional X-ray imaging, which involves a static, single-frame image.

Current established treatment strategies include activity modification, medications, physical therapy, postural rehabilitation, and upper cervical manual therapy. Emerging investigational treatments include prolotherapy (which involves injecting dextrose to stimulate a local inflammatory response and trigger ligamentous thickening and healing), PRP (which involves injecting the patient's own platelets to provide growth factors and initiate the healing cascade), PL (a filtered form of PRP with fewer particulates and more concentrated growth factors that is safer to use around spinal nerves), bone marrow concentrate (BMC), and occipito-cervical fusion for severe cases.

While occipito-cervical fusion can improve symptoms in appropriately selected patients, it is associated with permanent loss of motion and potential adjacent segment degeneration. Consequently, there is increasing interest in biologic treatments after conservative measures fail, which are intended to restore ligament competence while preserving native anatomy. Early clinical studies evaluating prolotherapy and platelet-based regenerative injections have demonstrated improvements in pain, function, and selected radiographic measures of cervical stability, although higher-quality prospective studies are still needed [6-9].

Historically, prolotherapy has been utilized for the treatment of ligamentous laxity and spinal instability. Early work by Hackett and Kayfetz suggested clinical improvement in patients with cervical and occipito-cervical instability treated with proliferant solutions, though these studies were limited by methodological constraints and the lack of imaging correlation [9-11]. More contemporary data from Centeno et al. demonstrated improvements in pain and radiographic stability following fluoroscopically guided cervical prolotherapy with blinded pre- and post-treatment imaging analysis, suggesting a potential structural effect beyond symptomatic relief [10].

More recent treatment paradigms emphasize the cervical spine as a functional spinal unit (FSU), wherein ligaments, facet joints, intervertebral discs, and nerves act in concert to maintain biomechanics. Dysfunction from structural or degenerative changes in one component may lead to compensatory changes and pain generation in adjacent segments. In this framework, regenerative therapies may be most effective when applied across multiple anatomical targets rather than to isolated structures, addressing both mechanical instability and downstream biomechanical consequences [10].

A major limitation in the interpretation of PRP literature is the significant heterogeneity in preparation techniques. PRP formulations vary widely in platelet concentration, leukocyte content, activation methods, and total injectate volume. These variables directly influence biologic activity, including growth factor release, inflammatory signaling, and tissue response. Furthermore, many studies fail to report total platelet dose, making comparisons across studies difficult and limiting the ability to establish dose-response relationships. This variability represents a critical barrier to standardization and may contribute to inconsistent clinical outcomes [12].

Interventions at the C0-C1 level present additional technical challenges and risk considerations. The atlanto-occipital joint is near critical neurovascular structures, including the vertebral artery, where an inadvertent injection may lead to significant morbidity and mortality, including a cerebral vascular incident or death. The small joint space and anatomic variability increase procedural complexity and the risk of complications such as vascular injury, dural puncture, or inadvertent brainstem injection. Fluoroscopic guidance with contrast confirmation is essential for safe needle placement. Centeno et al. described a fluoroscopically guided approach to the C0-C1 facet joint emphasizing these technical considerations and safety precautions [13]. Given these risks, many clinicians may not have adequate experience to perform this injection safely or may choose to treat lower cervical segments first (i.e., C2-C7 facet joints).

In patients with CCI, surgical stabilization via occipito-cervical fusion remains the definitive treatment when conservative measures fail. However, fusion is associated with significant risks, including neurologic injury, vascular complications, adjacent segment disease, and loss of physiologic motion [14]. In this context, regenerative therapies may serve as a minimally invasive alternative, with the potential to reduce symptoms, improve stability, and delay or avoid surgical intervention in appropriately selected patients.

## Conclusions

Regenerative injection therapies, including PRP and prolotherapy, may be a treatment option for patients with cervical spine pain and suspected instability, particularly within a functional spinal unit framework. This report highlights the importance of dynamic imaging in both diagnosis and treatment monitoring. Improvements in translational stability and alignment on serial DMX imaging suggest a possible structural effect of treatment, though causation cannot be definitively established in a single case study. Significant limitations of this study include the lack of objective outcome measures, observer bias, financial conflict of interest, possible placebo effect, and confounding variables, including natural disease variation and coinciding physical therapy/Egoscue, which could have impacted the treatment effect. Therefore, further research, including prospective controlled trials, should be performed to offer stronger statistical power, establish standardized protocols, define optimal patient selection, and clarify long-term outcomes.
